# Proteomic profiling reveals differentially expressed proteins associated with amylose accumulation during rice grain filling

**DOI:** 10.1186/s12864-020-07105-9

**Published:** 2020-10-15

**Authors:** Hengdong Zhang, Jiana Chen, Shuanglü Shan, Fangbo Cao, Guanghui Chen, Yingbin Zou, Min Huang, Salah F. Abou-Elwafa

**Affiliations:** 1 Crop and Environment Research Center, College of Agronomy, Hunan Agricultural University, Changsha, 410128 China; 2 Qianxinan Institute of Karst Regional Development Xingyi, Xingyi, 652400 Guizhou China; 3 Agronomy Department, Faculty of Agriculture, Assiut University, Assiut, 71526 Egypt

**Keywords:** Rice, Proteomic, Amylose, Glycolysis/gluconeogenesis, Starch and sucrose metabolism

## Abstract

**Background:**

Amylose accumulation in rice grains is controlled by genetic and environmental factors. Amylose content is a determinant factor of rice quality in terms of cooking and eating. Great variations in amylose content in *indica* rice cultivars have been observed. The current study was to identify differentially expressed proteins in starch and sucrose metabolism and glycolysis/gluconeogenesis pathways and their relationships to amylose synthesis using two rice cultivars possess contrasting phenotypes in grain amylose content.

**Results:**

Synthesis and accumulation of amylose in rice grains significantly affected the variations between rice cultivars in amylose contents. The high amylose content cultivar has three down-regulated differentially expressed proteins, i.e., LOC_Os01g62420.1, LOC_Os02g36600.1, and LOC_Os08g37380.2 in the glycolysis/gluconeogenesis pathway, which limit the glycolytic process and decrease the glucose-1-phosphate consumption. In the starch and sucrose metabolic pathway, an up-regulated protein, i.e., LOC_Os06g04200.1 and two down-regulated proteins, i.e., LOC_Os05g32710.1 and LOC_Os04g43360.1 were identified (Figure 4). Glucose-1-phosphate is one of the first substrates in starch synthesis and glycolysis that are catalyzed to form adenosine diphosphate glucose (ADPG), then the ADPG is catalyzed by granule-bound starch synthase I (GBSS I) to elongate amylose.

**Conclusions:**

The results indicate that decreasing the consumption of glucose-1-phosphate in the glycolytic process is essential for the formation of ADPG and UDPG, which are substrates for amylose synthesis. In theory, amylose content in rice can be regulated by controlling the fate of glucose-1-phosphate.

## Background

Rice is considered a staple food for more than half of the world’s population, therefore improving rice quality and productivity is essential to overcome the rapid population growth and meet the economic development and to ensure sustainable human food [1, 2]. Cooking and eating properties of rice grains are the main factors that influence consumer choice of preferred types of rice [3]. Amylose content is the key factor that affects cooking and eating quality of rice [4]. Therefore, the selection of rice cultivars with improved amylose content is of strategic importance in rice breeding programs [5].

Proteomics analysis is a direct and effective approach for identification of protein expression patterns and their post-translational modifications and has been applied to provide essential information for the differentiation of rice cultivars based on their protein contents [6, 7]. Besides, because of the relatively small genome size, employing proteomic profiling is an efficient and powerful approach in rice functional genomics in particular response to abiotic stresses such as high and low temperatures and salt stress [8–12].

Since the accumulation of amylose in rice grains is controlled by genetic and environmental factors, great variations in amylose content in *indica* rice cultivars ranged between 8.0–40.71% have been reported [5]. Amylose, a pivotal starch component, is a linear molecule composed of D-glucose units linked together by α-1,4 glycosidic bonds with occasional branching at α-1,6 branch points. However, the availability of adenosine diphosphate glucose (ADPG) as the substrate of amylose can limit amylose synthesis [13]. Genes and enzymes implicated in amylose synthesis are well-known and characterized. Amylose synthesis occurs in the pathways of starch and sucrose metabolism and glycolysis/gluconeogenesis and is directly linked to starch and sucrose metabolism [14]. Amylose synthesis is governed by adenosine diphosphoglucose (ADP-glucose) pyrophosphorylase that is catalyzed by the waxy gene encoded protein granule-bound starch synthase I (GBSS I) that affects cooking and eating quality attributes of rice [13, 15, 16]. There are two functional *waxy* alleles, i.e., *Wx*^*a*^ and *Wx*^*b*^. The *Wx*^*a*^ allele is mainly distributed in the *indica* genotypes and is located to chromosome 6 [17, 18]. The enzyme GBSS underlies the accumulation of amylose in rice grains [18]. However, there has been limited research on the differentially expressed proteins related to amylose synthesis that are also implicated in the pathways of glycolysis/gluconeogenesis and starch and sucrose metabolism.

In this study, we have selected two rice cultivars exhibited contrasting amylose content levels to identify the differentially expressed proteins in the pathways of glycolysis/gluconeogenesis and starch and sucrose metabolism and to identify their relationships with amylose synthesis.

## Results

### Grain and amylose parameters

**Fig. 1:**
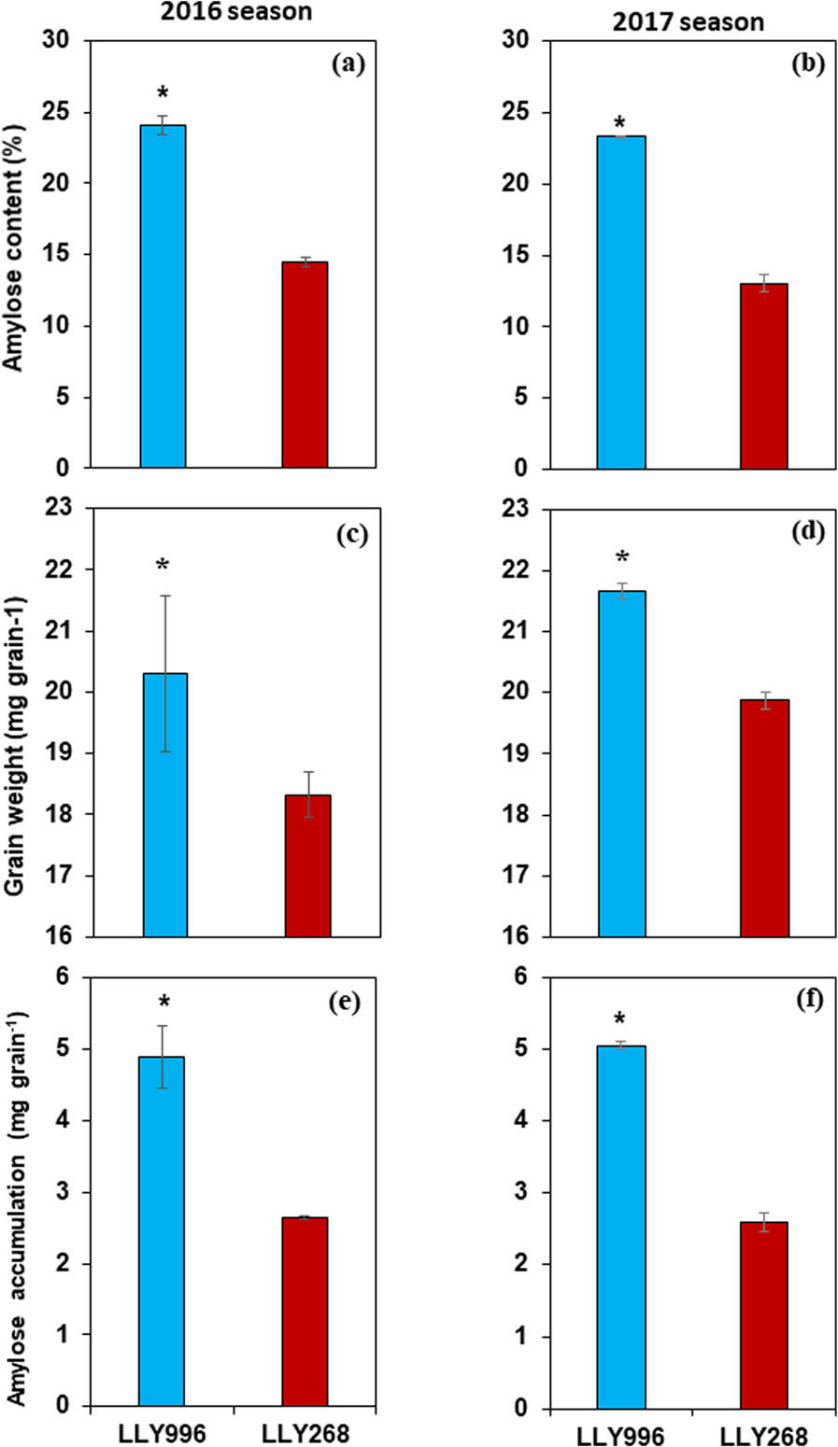
Amylose content (**a** & **b**), grain weight (**c** & **d**) and amylose accumulation (**e** & **f**) at grain maturity of rice cultivars LLY996 (Luliangyou 996) and LLY268 (Lingliangyou 268) in 2016 and 2017 growing seasons. Error bars represent SD, * indicates significance at 0.05 level between the two rice cultivars

**Fig. 2:**
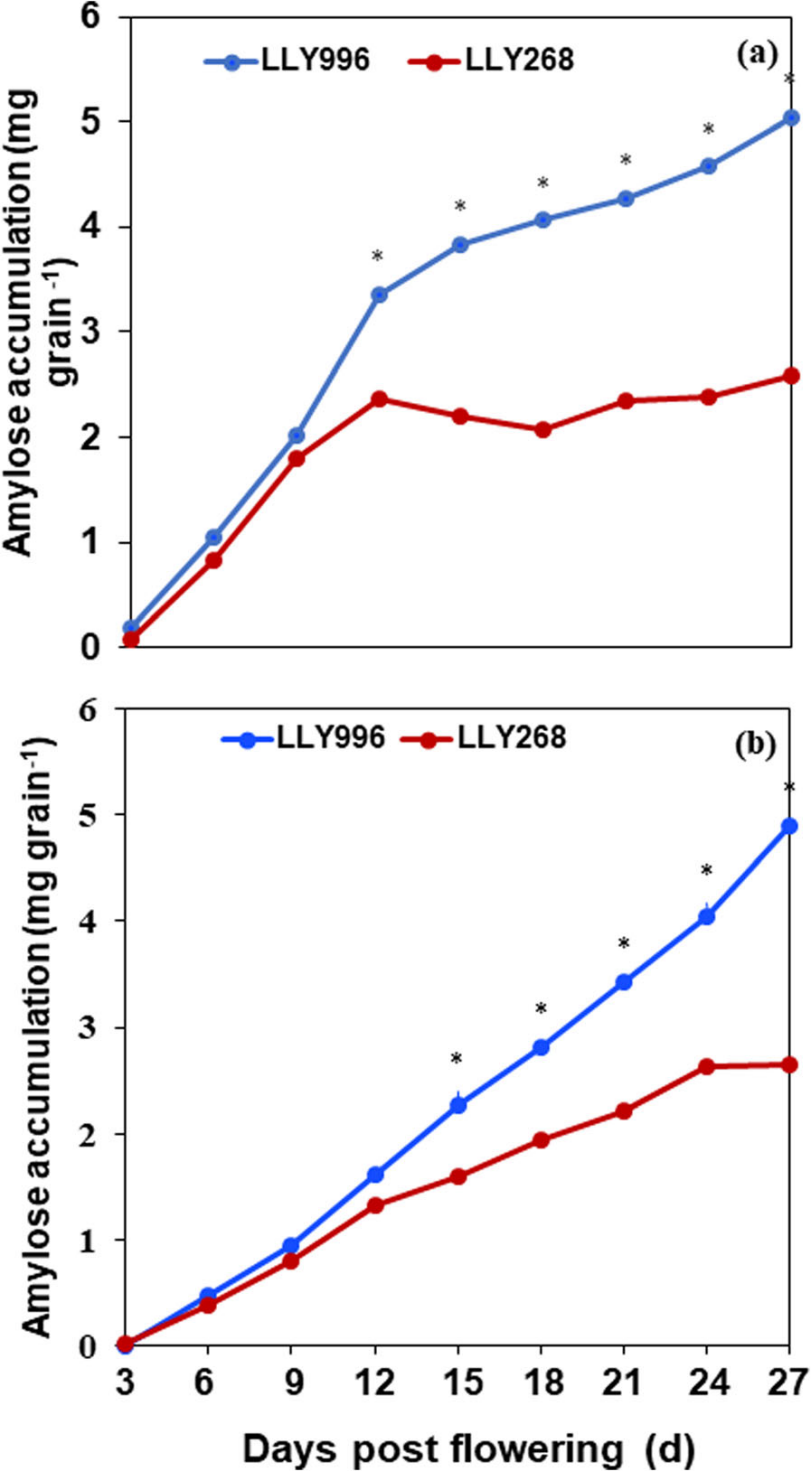
Amylose accumulation during the grain filling stage in 2016 (**a**) and 2017 (**b**) growing seasons. * indicates significant differences (*P* < 0.05). LLY996: Luliangyou 996 and LLY268: Lingliangyou 268

Differences in amylose accumulation between the two rice cultivars increased over time post-flowering. Within the first 9 days post-flowering, the accumulation of amylose did not differ significantly between the two cultivars. Meanwhile, from the ninth day post-flowering until grain maturity, amylose accumulation was significantly increased in the cultivar LLY996 compared to the cultivar LLY268 (Fig. 1). The data revealed that the cultivar LLY996 surpassed the cultivar LLY268 in grain-filling and amylose accumulation rates over the two growing seasons (Fig. 2). The grain-filling and amylose accumulation processes were both well fitted by the logistic equation for both cultivars. The grain filling process exhibited highly significant determination coefficients (R^2^) of 0.981 and 0.983, and 0987 and 0.988 for LLY996 and LLY268, in the first and second growing seasons, respectively. Likewise, the amylose accumulation process revealed significant determination coefficients (*R*^2^) of 0.980 and 0.969, and 0987 and 0.984 for LLY996 and LLY268, in the first and second growing seasons, respectively (Table 1). At maturity, amylose content was significantly higher (> 70%) in the cultivar LLY996 than the cultivar LLY268 in both growing seasons (Table 1). Grain weight and amylose accumulation were respectively 9 and 10%, and 95 and 93% higher in the cultivar LLY996 than the cultivar LLY268 in the first and second growing seasons, respectively (Fig. 1; Table 2).

**Table 1:** The fitted logistic equation and parameters of amylose accumulation with the two rice varieties in 2016 and 2017 growing seasons

Year	Variety	Fitted equation	Correlation coefficient R^2^	accumulation rate (mg grain^− 1^ d^− 1^)						The maximum amylose accumulation rate		Amylose accumulation duration (d)			
				Initial	Maximum	Mean	Gradual	Rapid	Slowly	Time (d)	Accumulation (mg grain^−1^ d^−1^)	Gradual	Rapid	Slowly	Sum
2016	LLY996	Y = 4.62/(1 + EXP(3.27–0.335X))	0980**	0.055	0.387	0.197	0.17	0.34	0.14	9.8	2.33	5.8	7.9	3.9	17.6
	LLY268	Y = 2.34/(1 + EXP(4.72–0.672X))	0.969**	0.014	0.393	0.169	0.10	0.35	0.15	7.0	1.16	5.1	3.9	1.8	10.8
2017	LLY996	Y = 5.59/(1 + EXP(3.25–0.182X))	0987**	0.037	0.254	0.130	0.11	0.22	0.11	17.9	2.81	10.6	14.5	7.2	32.3
	LLY268	Y = 2.74/(1 + EXP(3.06–0.230X))	0.984**	0.027	0.158	0.082	0.08	0.14	0.07	13.3	1.37	7.6	11.5	5.7	24.8

**Table 2:** The fitted logistic equation and parameters during grain filling-stage of the two rice varieties in 2016 and 2017 growing seasons

Year	Variety	Fitted equation	Correlation coefficient R^2^	Grain filling rate (mg grain^−1^ d^−1^)						The maximum rate		The grain-filling duration			
				Initial	Maximum	Mean	Gradual	Rapid	Slowly	Time (d)	Accumulation (mg grain^−1^ d^−1^)	Gradual	Rapid	Slowly	Sum
2016	LLY996	Y = 20.14/(1 + EXP(2.41–0.299X))	0.981**	0.455	1.505	0.860	1.16	1.32	0.65	8.1	10.13	3.7	8.8	4.5	17.0
	LLY268	Y = 18.89/(1 + EXP(1.71–0.395X))	0.983**	0.909	1.752	1.112	3.75	1.61	0.65	4.6	9.95	1.1	7.1	3.8	11.9
2017	LLY996	Y = 21.40/(1 + EXP(2.93–0.210X))	0987**	0.216	1.124	0.597	0.59	0.99	0.49	14.0	10.75	7.7	12.5	6.4	26.6
	LLY268	Y = 18.11/(1 + EXP(2.87–0.245X))	0.988**	0.225	1.109	0.594	0.60	0.97	0.49	11.7	9.04	6.3	10.8	5.4	22.5

### Protein identification and GO enrichment analysis

**Fig. 3:**
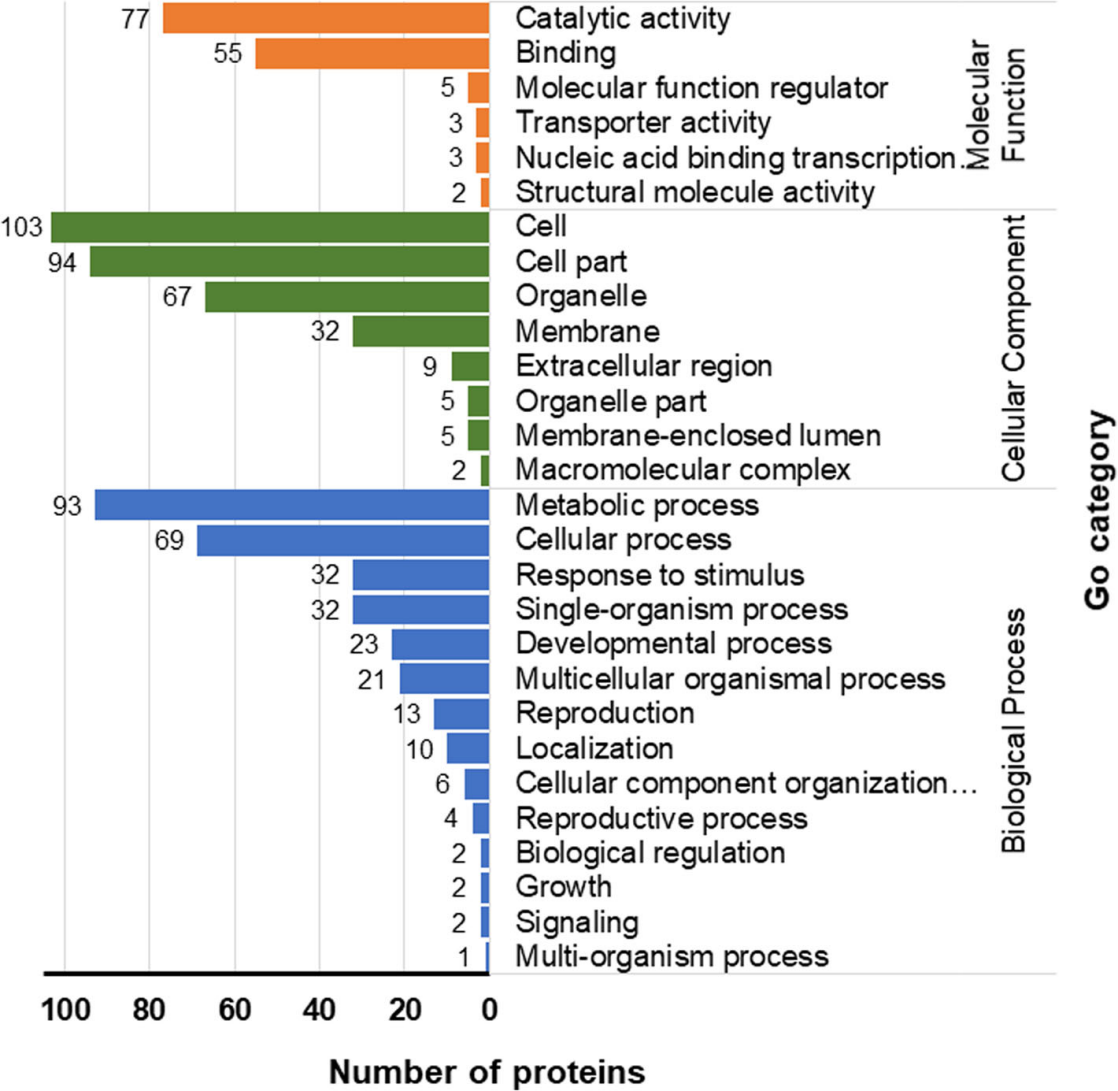
Statistical distribution chart of differentially expressed proteins under each GO category (2nd level)

To dissect molecular mechanisms underlying amylose accumulation in rice grains, the iTRAQ approach coupled with LC-MS/MS was employed to analyze grains proteomics of the two rice cultivars LLY996 and LLY268 at 12 days post-flowering. Quality control filtering revealed a total of 3634 highly reproducible proteins that could be quantified in the LLY996 and LLY268 cultivars. Proteomic profiling exhibited 149 differentially expressed proteins in grains between LLY996 and LLY268 at 12 days post-flowering. The number of amino acids of those proteins ranged between 78 and 1431, and their molecular weight ranged between 8.70–152.30 kDa. Thirty-three out of those 149 differentially abundant proteins were up-regulated while the remaining 133 proteins were down-regulated (Table S1). Gene Ontology (GO) classified deferentially expressed proteins into 28 GO classification groups including biological process (BP), cellular compartment (CC) and molecular function (MF). Among these GO groups, six were molecular functions, eight were cellular components and 14 were biological processes (Table S2). The BP group comprises metabolic processes, single-organism processes and cellular processes. Proteins with differential expression levels of the CC category are mostly involved in cellular component organization or biogenesis, membrane and extracellular region. The most prevalent proteins in the MF group comprises membrane and organelle, catalytic activity and transporter activity (Fig. 3).

### KEGG annotations of deferentially expressed proteins

The Kyoto Encyclopedia of Genes and Genomes (KEGG) pathway enrichment analysis assigned the 149 differentially expressed proteins into 58 metabolic pathways (Table S3). According to the KEGG analysis, the Starch and sucrose metabolism pathway that involves the process of amylose synthesis and the Glycolysis/gluconeogenesis pathway is directly linked to starch and sucrose metabolism. There was six differentially expressed proteins involved in these two metabolic pathways, from which one is up-regulated protein and five are down-regulated proteins. The up-regulated protein in addition to two of the five down-regulated proteins are involved in starch and sucrose metabolism, while the other three down-regulated proteins are involved in glycolysis/gluconeogenesis (Table 3).

**Table 3:** Differentially expressed proteins identified in the glycolysis/gluconeogenesis and starch and sucrose metabolism pathways

Pathway	Accession	Description	FC
Glycolysis/ Gluconeogenesis	LOC_Os01g62420.1	triosephosphate isomerase, cytosolic, putative, expressed	0.826
	LOC_Os02g36600.1	aldose 1-epimerase, putative, expressed	0.725
	LOC_Os08g37380.2	glucose-6-phosphate isomerase, putative, expressed	0.662
Starch and sucrose metabolism	LOC_Os06g04200.1	starch synthase, grand-bound starch synthase 1, chloroplastic/ amyloplastic	3.39
	LOC_Os05g32710.1	isoamylose 2, chaloroplastic, putative,expressed	0.789
	LOC_Os04g43360.1	Os4bglu14 - monolignol beta-glucosidase homologue without catalytic acid/base, expressed	0.784

Note: FC (fold change) for Luliangyou 996 (a high amylose content rice variety)/Lingliangyou 268 (a low amylose content rice variety); proteins abundances with FC>1.2 or FC<0.833 (*p*<0.05) were considered up-regulated or down-regulated, respectively

## Discussion

Proteomics profiling has been approved as a powerful molecular strategy that has been widely implemented in dissecting the molecular basis of various biological processes in living organisms including plants. However, the experimental system and procedure of the employed proteomics profiling approach greatly affect the power and efficiency of proteomic profiling in dissecting the molecular mechanisms of a biological process [19–28]. In rice, seed development is a complex biological process that greatly affects grain yield and quality and is governed by complex regulatory networks comprising numerous transcription factors [29].

The current study has been carried out to uncover proteins implicated in amylose accumulation during the early period of grain filling in rice. Two rice cultivars, i.e., LLY996 than in LLY268, differed greatly in amylose accumulation during grain filling and in amylose content of mature grains were employed in the identification of differentially expressed proteins that might be implicated in amylose accumulation during grain filling. The data revealed that the cultivar LLY996 surpassed the cultivar LLY268 in grain-filling and amylose accumulation rates over the two growing seasons. Besides, amylose accumulation was significantly increased from the ninth day post-flowering until grain maturity in the cultivar LLY996 compared to the cultivar LLY268. These findings are consistent with previous results where a significant increment of amylose accumulation occurred primarily at 5–15 days post-flowering [30] and the highest rate of amylose accumulation occurred at 3–12 days post-flowering [31], indicating the suitability of grain samples collected 12 days post-flowering for the identification of proteins implicated in amylose accumulation during grain filling. Besides, the grain-filling and amylose accumulation processes were both well-fitted by the logistic equation for both cultivars, demonstrating the appropriateness of the two selected cultivars for quantitative proteomic profiling. The higher grain weight observed in the cultivar LLY996 compared to the cultivar LLY268 at maturity could be due to the higher-yielding ability of the cultivar LLY996 compared to the cultivar LLY268 (Fig. S1) which is due to the genetic composition of the two cultivars (Fig. 1).

In starch and sucrose metabolism, the enzyme isoamylase (ISA, EC: 3.2.1.68) is a starch debranching enzyme that has three isoforms, i.e., ISA1, ISA2 and ISA3, two of which, i.e., ISA1 and ISA3, are strongly implicated in amylopectin synthesis. Furthermore, although the ISA2 isoform appears to be catalytically inactive, it may modulate the action or stability of ISA1 [32]. However, all three isoforms reduce granular starch, where amylose synthesis occurs within the granules [32, 33]. In our study, the differentially expressed protein LOC_Os05g32710.1 (ISA2) was down-regulated in the cultivar LLY996 and up-regulated in the cultivar LLY268 (Table 3), which is similar to a previous report of ISA as a starch debranching enzyme that has been up-regulated in a low-amylose content rice mutant [14]. The accumulation rate of amylose is positively correlated with the activity of the Granule-bound starch synthase (GBSS) enzyme [34]. A similar relationship was observed in our study where the protein associated with LOC_Os06g04200.1 which is involved in GBSS activity has been up-regulated (3.39-fold change) in the cultivar LLY996 compared to the cultivar LLY268 (Table 3). These findings suggest the implication of the locus LOC_Os06g04200.1 in enhancing amylose synthesis and accumulation in rice.

Several differentially abundant proteins have been identified to be implicated in the glycolysis and gluconeogenesis which involve reversed biochemical reactions of each other’s pathways and most of the associated enzymes take part in reversible reactions of the pathways [14, 35]. Glucose-6-phosphate isomerase (EC: 5.3.1.9) catalyzes the glucose-6-phosphate and fructose-6-phosphate and the reaction is reversible [36]. Triosephosphate isomerase (EC: 5.3.1.1) is involved in sugar metabolism and, basically, the pathway of glycolytic synthesis of ATP [37]. The aldose-1- epimerase protein is the key enzyme (EC: 5.1.3.3) of carbohydrate metabolism and catalyzes the interconversion of α- and β-anomers of sugar [38]. The key enzyme 6-phosphofructokinase, which is a pyruvate kinase and pyruvate phosphate dikinase catalyze irreversible reactions in glycolysis [14], did not show differential expression between the two cultivars, suggesting that there was no Gluconeogenesis involved during grain filling and amylose accumulation. There was three differentially expressed proteins, i.e., LOC_Os01g62420.1, LOC_Os02g36600.1, and LOC_Os08g37380.2, which exhibited 0.826, 0.725, and 0.662 fold changes in the cultivar LLY996 compared to the cultivar LLY268 and are known to be implicated in triosephosphate isomerase, aldose-1- epimerase, and glucose-6-phosphate isomerase, respectively (Table 3). These three enzymes limit the glycolytic process and decreased the glucose-1-phosphate consumption.

**Fig. 4:**
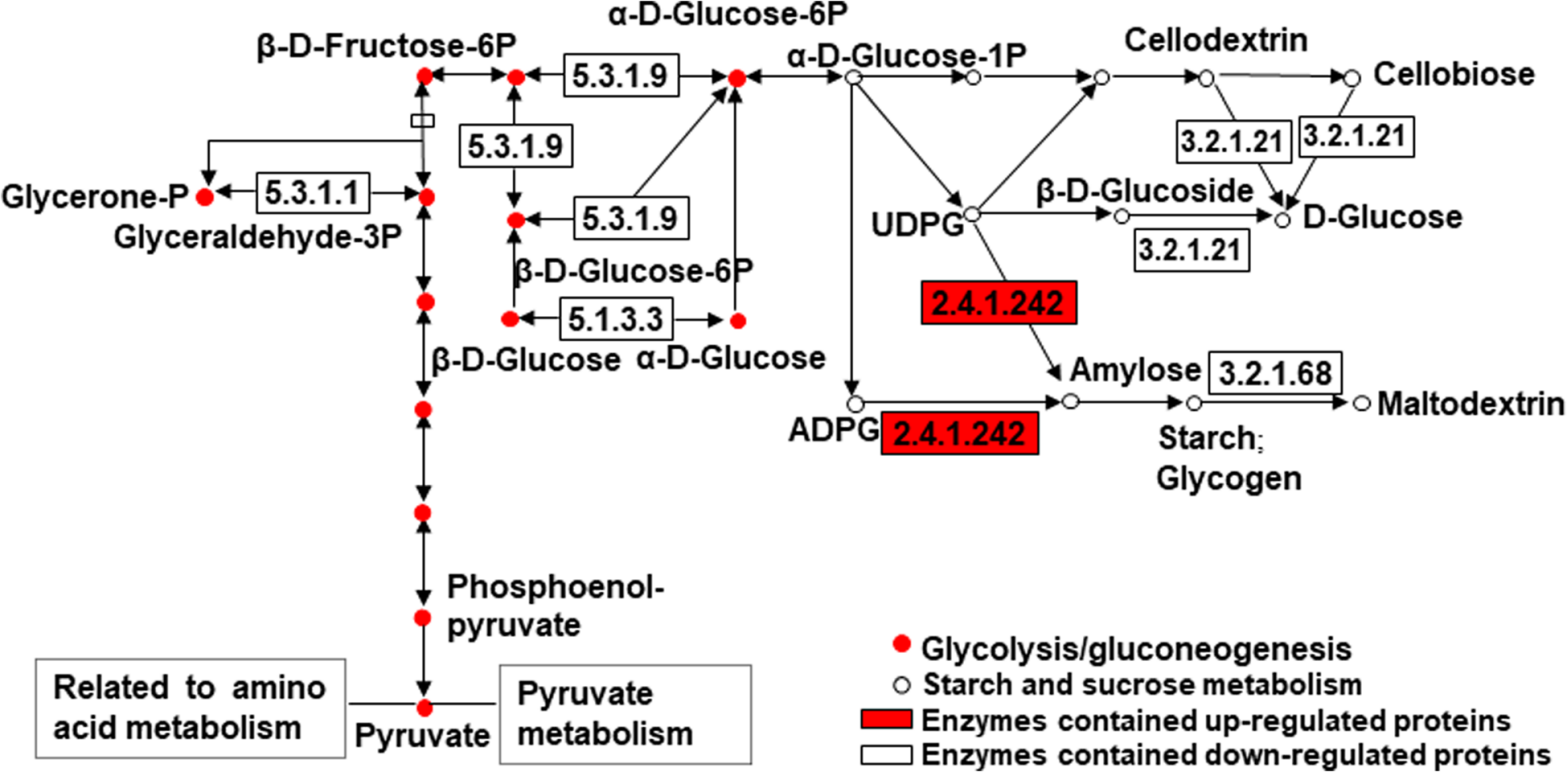
Differentially expressed proteins identified in pathways of “glycolysis/gluconeogenesis” and “starch and sucrose metabolism” with Luliangyou 996 (a high amylose content rice cultivar)/Lingliangyou 268 (a low amylose content rice cultivar). Only the enzymes with differentially expressed proteins and their substrates and products are shown

Glucose-1-phosphate is a key factor that links glycolysis/gluconeogenesis and starch and sucrose metabolism (Fig. 4). Glucose-1-phosphate is one of the first substrates in starch synthesis and glycolysis [39, 40]. It is one of the substrates that are catalyzed to form adenosine diphosphate glucose (ADPG), then the ADPG is catalyzed by GBSS to elongate amylose [13, 14]. In our study, we hypothesized that uridine diphosphate glucose (UDPG) obtained single glucose from glucose-1-phosphate and then was catalyzed by GBSS to form amylose. Reportedly, UDPG could be converted into hexose phosphates and take on roles in starch synthesis [40, 41]. In the process of amylose synthesis, maltohexose acts as one form of primers in plants [16]. However, what still remains to be investigated is whether UDPG provides the hexose or, similar to the role of ADPG, provides the single glucose molecule for the primer to elongate amylose. The use ratio of glucose-1-phosphate in starch and sucrose metabolism and glycolysis also has to be further studied.

Glycolysis and gluconeogenesis involve nearly reversed biochemical reactions of each other’s pathways [35] and most of the associated enzymes take part in reversible reactions of the pathways [14]. Glucose-6-phosphate isomerase (EC: 5.3.1.9) catalyzes the glucose-6-phosphate and fructose-6-phosphate and the reaction is reversible [36]. Triosephosphate isomerase (EC: 5.3.1.1) is involved in sugar metabolism and, basically, the pathway of glycolytic synthesis of ATP [37]. The aldose-1- epimerase protein is the key enzyme (EC: 5.1.3.3) of carbohydrate metabolism and catalyzes the interconversion of α- and β-anomers of sugar [38]. The key enzymes 6- phosphofructokinase, pyruvate kinase and pyruvate phosphate dikinase catalyze irreversible reactions in glycolysis [14] and we did not observe differences in the expression of any of these enzyme between the two cultivars, which suggests there was no Gluconeogenesis occurring.

## Conclusions

Amylose accumulation results showed significant differences between different rice cultivars with different amylose contents at the 12th DPF to maturity in grains. We analyzed the differentially expressed proteins from grain sampled at the 12th DPF. The DEP LOC_Os06g04200.1 (granule bound starch synthase I) was 3.39-fold up-regulated in the cultivar LLY996 compared to the cultivar LLY268, suggesting that this protein is crucial for the accumulation of amylose in grains. There were three enzymes, i.e., triosephosphate isomerase, aldose-1-epimerase, and glucose-6-hosphate isomerase which respectively contained three down-regulated differentially expressed proteins, i.e., LOC_Os01g62420.1, LOC_Os02g36600.1, and LOC_Os08g37380.2. The glycolytic process in the cultivar LLY996 was likely limited by these three enzymes and decreased glucose-1-phosphate consumption. Our results indicate that the decreasing the consumption of glucose-1-phosphate is crucial for the synthesis of ADPG and UDPG which are essential substrates for amylose synthesis and that UDPG plays an important role as one of the substrates in amylose synthesis. However, future studies implementing variable rice genotypes for validation of these results and for better understanding of the importance of this study are necessary.

## Methods

### Plant materials and experiments

Two *indica* rice cultivars, i.e., Luliangyou 996 (LLY996) and Lingliangyou 268 (LLY268), provided by the Hunan Rice Research Institute, Changsha, China exhibiting contrasting phenotypes in amylose content were used in the current study. The cultivar LLY996 has a high grain amylose content of up to 24.2%, while the LLY268cultivar has a low grain amylose content (12.3%). Field experiments were carried out during two successive growing seasons in 2016 and 2017 in Yongan Town, Hunan Province, China (28°09′ N, 113°37′ E, 43 m asl). The climatic data of the experimental site during the grain filling period in 2016 and 2017 growing seasons are presented in Table S4. We comply with the Convention on the Trade in Endangered Species of Wild Fauna and Flora (https://www.cites.org/). Soil samples collected from the 0–20 cm surface layer prior to the beginning of the experiment in 2016 were used for the physical and chemical analyses of the experimental site soil. The basic physical and chemical characteristics of the experimental field soil are shown in Table 4. The experimental design of the experiments followed the in a randomized complete block design in three replications with an experimental unit (plot) size of 40 m^2^. Seeds were sown on March 29, 2016, in trays. The high-speed rice transplanter (PZ80–25, Dongfeng Iseki Agricultural Machinery Co., Ltd., Xiangyang, China) were implemented to transplant the 25-days-old seedlings into the field on April 20 at 25 cm spacing between rows and 11 cm between plants within rows. Fertilizers were applied in the ratio of 2:1:2, N: P_2_O_5_: K_2_O. A total amount of nitrogen fertilizer at 135 kg ha^− 1^ rate was applied in three doses, i.e., 50% as a basal fertilization dose applied a day before transplanting, 20% as tillering fertilization dose applied 7 days post-transplanting, and 30% as a head-dressing dose. Phosphorus fertilization of 67.5 kg P_2_O_5_ ha^− 1^ was applied as a basal fertilization dose, while potassium fertilization of 135 kg K_2_O ha^− 1^ was applied into two doses, i.e., 50% as a basal dose and 50% as a head dressing dose.

**Table 4:** Basic physical and chemical properties of the experimental field soil prior to the beginning of the experiment in 2016

Soil type	pH	Organic matter (mg kg^− 1^)	Avilable N (mg kg^− 1^)	Avilable P (mg kg^− 1^)	Avilable K (mg kg^− 1^)
Clay	6.07	36.18	203.17	16.02	190.19

### Sampling and protein extraction

A total of 120 panicles flowered in the same day from each plot were tagged and designated as the day 1 post-flowering (1DPF). From the third-day post-flowering (3DPF), 10 tagged panicles were randomly sampled every 3 days until rice grains reached maturity. Half of the collected samples were oven-dried at 70 °C to a constant dry-weight and their seeds were removed and hulled by hand for grain amylose content determination. Amylose content was determined using iodine-blue colorimetry. The other half of the tagged panicle samples were frozen in liquid nitrogen and kept at − 80 °C for total protein extraction using the acetone procedure [42].

### Fractionation and identification of proteins

For proteomics profiling, grains sampled from plants at 12 DPF were used. Fractionation and identification of tryptic peptides were performed using the iTRAQ (isobaric tags for relative and absolute quantitation) approach coupled with LC-MS/MS (liquid chromatography-mass spectrometry/ mass spectrometry). In brief, for fractionation of tryptic peptides, the Agilent 300Extend C18 column in the high pH reverse-phase HPLC was used. Fractionated peptides were then grouped into 18 fractions and vacuum-centrifuged till dry. The tryptic peptides were then dissolved in 0.1% formic acid and loaded onto a reversed-phase analytical column (15-cm length, 75 μm i.d.). The Q ExactiveTM Plus (Thermo Fisher Scientific, Waltham, MA, USA) was employed in tandem mass spectrometry (MS/MS). The m/z scan range was at 350 to 1800 for a full scan, and the Orbitrap was then implemented to identify the intact peptides at a resolution of 70,000. Peptides MS/MS was carried out using the NCE setting as 28, the Orbitrap was employed to identify the fragments at a resolution of 17,500. Automatic gain control (AGC) was set to 5E4, and the fixed first mass was set to 100 m/z. The Maxquant search engine (v.1.5.2.8) was employed in processing the resulting MS/MS data.

The raw mass data were processed for the peptide data analysis using Proteome Discoverer 1.4 (ver.1.4.0.288, Thermo Fisher Scientific) with a false discovery rate (FDR) < 1% and expected cutoff or ion score < 0.05 (with 95% confidence) in the search through the Rice MSU database (http://rice.plantbiology.msu.edu/). The fold change (FC) of DEPs in rice grains was calculated as the ratio of protein abundances of LLY996/LLY268. The value of FC was used to indicate whether a protein was significantly (*p*<0.05) up- (FC>1.20) or down-regulated (FC<0.833). Gene Ontology (GO) (http://www.geneontology.org,) proteome annotation was performed on the differentially abundant proteins to identify their molecular functions. The Kyoto Encyclopedia of Genes and Genomes (KEGG) database (http://www.kegg.jp/kegg/pathway.html/) was employed to determine the interactions among these proteins in terms of the biological pathways.

### Statistical analysis

Analysis of variance (ANOVA) and least significant difference (LSD) in the Statistix 8.0 software (Tallahassee, FL, USA) were employed to analyze amylose content and accumulation and grain weight. The SigmaPlot 14 Software (Systat Software, San Jose, CA, USA) was implemented to perform linear regression coefficients (R) of measured traits.

## Supplementary information

**Additional file 1: Table S1**. Identified differentially expressed proteins in Luliangyou 996 compared to Lingliangyou 268. (XLS 100 kb)

**Additional file 2: Table S2**. Gene Ontology (GO) enrichment analysis of differentially expressed proteins. (XLS 105 kb)

**Additional file 3: Table S3**. Kyoto Encyclopedia of Genes and Genomes (KEGG) pathway enrichment analysis of differentially expressed proteins. (XLS 46 kb)

**Additional file 4: Table S4**. The average, maximum and minimum temperatures and incident radiation during grain filling period in 2016 and 2017 growing seasons.

## Data Availability

All data are included within the manuscript and its supplementary material.

## Abbreviations

FDR: False discovery rate
AGC: Automatic gain control
DPF: Days post flowering
DEP: Differentially expressed proteinsADPG: adenosine diphosphate glucose
GBSS: Granule-bound starch synthase
UDPG: Uridine diphosphate glucose
ISA: Isoamylase enzyme
R^2^: Determination coefficient
GO: Gene Ontology
BP: Biological process
CC: Cellular compartment
MF: Molecular function
KEGG: The Kyoto Encyclopedia of Genes and Genomes
ANOVA: Analysis of variance
