# Supplementary material for: Proteomic profiling reveals differentially expressed proteins associated with amylose accumulation during rice grain filling
Source: BMC Genomics. 2020 Oct 15;21:714. doi: 10.1186/s12864-020-07105-9 (PMC7561244; doi:10.1186/s12864-020-07105-9)
Supplement: Supplementary file 4 — Additional file 4: Table S4. The average, maximum and minimum temperatures and incident radiation during grain filling period in 2016 and 2017 growing seasons. [file 12864_2020_7105_MOESM4_ESM.docx]

Table S4. The average, maximum and minimum temperatures and incident radiation during grain filling period in 2016 and 2017 growing seasons.

| 2016 | | | | | 2017 | | | | | | |
| --- | --- | --- | --- | --- | --- | --- | --- | --- | --- | --- | --- |
| Days after heading | Temperature (℃） | | | Incident radiation | Days after heading | Temperature (℃） | | | | | Incident radiation |
|  | Mean | Max | Min | (MJ m^-2^) |  | Mean | Max | | Min | | (MJ m^-2^) |
| 6/19/2016 | 29.9 | 33.3 | 26.4 | 19.01 | 6/21/2017 | 24.87 | 28.40 | | 22.30 | | 9.69 |
| 6/20/2016 | 29.6 | 31.8 | 27.5 | 12.67 | 6/22/2017 | 26.82 | 33.10 | | 21.70 | | 16.52 |
| 6/21/2016 | 30.7 | 34.5 | 27.3 | 20.82 | 6/23/2017 | 25.38 | 26.40 | | 24.40 | | 3.71 |
| 6/22/2016 | 31.3 | 35.8 | 27 | 24.93 | 6/24/2017 | 23.94 | 26.30 | | 22.20 | | 1.60 |
| 6/23/2016 | 31.5 | 35.1 | 26.8 | 27.49 | 6/25/2017 | 23.33 | 24.70 | | 22.10 | | 5.81 |
| 6/24/2016 | 27.6 | 33.4 | 22.2 | 21.53 | 6/26/2017 | 23.60 | 25.90 | | 22.40 | | 6.22 |
| 6/25/2016 | 20.6 | 22.4 | 19.6 | 4.21 | 6/27/2017 | 22.65 | 25.90 | | 21.00 | | 9.28 |
| 6/26/2016 | 23.2 | 28.5 | 19.6 | 14.71 | 6/28/2017 | 23.20 | 26.40 | | 21.40 | | 8.93 |
| 6/27/2016 | 23.2 | 25.2 | 22.3 | 6.68 | 6/29/2017 | 23.48 | 24.70 | | 22.30 | | 4.44 |
| 6/28/2016 | 23.8 | 29.4 | 20.2 | 18.05 | 6/30/2017 | 23.79 | 25.60 | | 22.30 | | 2.26 |
| 6/29/2016 | 25.1 | 29.1 | 22.9 | 8.5 | 7/1/2017 | 23.65 | 24.90 | | 22.80 | | 2.01 |
| 6/30/2016 | 27.9 | 31.4 | 25 | 13.55 | 7/2/2017 | 26.01 | 29.60 | | 23.50 | | 14.61 |
| 7/1/2016 | 27.5 | 29.1 | 26.1 | 7.02 | 7/3/2017 | 26.53 | 30.70 | | 23.60 | | 19.22 |
| 7/2/2016 | 25.8 | 28.6 | 24.7 | 8.15 | 7/4/2017 | 27.53 | 31.70 | | 24.30 | | 15.41 |
| 7/3/2016 | 25 | 26.4 | 24.2 | 4.59 | 7/5/2017 | 29.16 | 33.20 | | 25.40 | | 20.38 |
| 7/4/2016 | 23.8 | 25.3 | 22.2 | 2.62 | 7/6/2017 | 29.89 | 33.80 | | 25.70 | | 22.77 |
| 7/5/2016 | 24.3 | 29 | 22.4 | 10.37 | 7/7/2017 | 30.50 | 35.80 | | 27.00 | | 22.48 |
| 7/6/2016 | 24.9 | 29.6 | 22.2 | 16.93 | 7/8/2017 | 29.32 | 31.50 | | 27.30 | | 14.25 |
| 7/7/2016 | 27.6 | 34.1 | 23.6 | 23.1 | 7/9/2017 | 28.56 | 31.70 | | 26.20 | | 9.58 |
| 7/8/2016 | 29.1 | 35 | 25.4 | 18.97 | 7/10/2017 | 25.57 | 27.60 | | 23.80 | | 8.07 |
| 7/9/2016 | 30.5 | 36 | 25.3 | 27.03 | 7/11/2017 | 28.03 | 30.80 | | 24.30 | | 14.21 |
| 7/10/2016 | 29.6 | 34.3 | 25.9 | 20.27 | 7/12/2017 | 30.08 | 35.80 | | 25.10 | | 22.64 |
| 7/11/2016 | 30.1 | 34.7 | 26.6 | 25.73 | 7/13/2017 | 31.22 | 36.10 | | 27.30 | | 20.62 |
| 7/12/2016 | 29.8 | 36.1 | 26.3 | 22.41 | 7/14/2017 | 31.48 | 36.10 | | 27.00 | | 22.57 |
| 7/13/2016 | 28.6 | 34.2 | 26.3 | 13.21 | 7/15/2017 | 31.91 | 37.10 | | 25.90 | | 25.04 |
| 7/14/2016 | 28.5 | 34.2 | 25.9 | 17.99 | 7/16/2017 | 32.70 | 38.20 | | 26.70 | | 25.07 |
| 7/15/2016 | 28.3 | 33.6 | 25 | 17.28 | 7/17/2017 | 33.00 | 38.70 | | 27.10 | | 22.07 |
| 7/16/2016 | 26.8 | 30.3 | 24.5 | 12.11 | 7/18/2017 | 31.50 | 35.20 | | 27.40 | | 17.63 |
| 7/17/2016 | 27.4 | 29.1 | 25.9 | 6.89 |  |  | |  | |  |  |
